# Medical Legislation in Illinois

**Published:** 1869-03-01

**Authors:** 


### *Medical Legislation in Illinois.*

At the present writing it would appear that the very absurd act for the protection (!) of the medical profession in this State, has received its quietus at the hands of the Legislature.

The very desirable statute for the regulation and promotion of the study of Anatomical Science, has passed the House, and has been ordered to a third reading in the Senate, which we suppose is about equivalent to its passage. The profession and the public are to be congratulated at the result.

Some one or more Sorrows-of-Werter Sentimentalists in the Senate, who should have been born in the Middle Ages, opposed the bill in their most forcibly-feeble manner, but the good sense of the body triumphed. *Laus Senatu!*

